# The application of multi-baseline digital close-range photogrammetry in three-dimensional imaging and measurement of dental casts

**DOI:** 10.1371/journal.pone.0178858

**Published:** 2017-06-22

**Authors:** Xiaoming Fu, Chun Peng, Zan Li, Shan Liu, Minmin Tan, Jinlin Song

**Affiliations:** 1 Chongqing Key Laboratory for Oral Diseases and Biomedical Sciences, Chongqing, China; 2 College of Stomatology, Chongqing Medical University, Chongqing, China; 3 Chongqing Municipal Key Laboratory of Oral Biomedical Engineering of Higher Education, Chongqing, China; 4 Yongchuan Hospital of Chongqing Medical University, Chongqing, China; 5 Department of Pathology, Chongqing People 's Hospital, Chongqing, China; Monash University, AUSTRALIA

## Abstract

**Objective:**

To explore a new technique for reconstructing and measuring three-dimensional (3D) models of orthodontic plaster casts using multi-baseline digital close-range photogrammetry (MBDCRP) with a single-lens reflex camera.

**Study design:**

Thirty sets of orthodontic plaster casts that do not exhibit severe horizontal overlap (>2 mm) between any two teeth were recorded by a single-lens reflex camera with 72 pictures taken in different directions. The 3D models of these casts were reconstructed and measured using the open source software MeshLab. These parameters, including mesio-distal crown diameter, arch width, and arch perimeter, were recorded six times on both the 3D digital models and on plaster casts by two examiners. Statistical analysis was carried out using the Bland–Altman method to measure agreement between the novel method and the traditional calliper method by calculating the differences between mean values.

**Results:**

The average differences between the measurements of the photogrammetric 3D models and the plaster casts were 0.011–0.402mm. The mean differences between measurements obtained by the photogrammetric 3D models and the dental casts were not significant except for the lower arch perimeter (*P*>0.05), and all the differences were regarded as clinically acceptable (<0.5 mm).

**Conclusions:**

Measurements obtained by MBDCRP are compared well with those obtained from plaster casts, indicating that MBDCRP is an alternate way to store and measure dental plaster casts without severe horizontal overlap between any two teeth.

## Introduction

Orthodontic plaster casts play an important role in the diagnosis of malocclusion, the planning of treatments, and the evaluation of treatment outcomes. They also serve as carriers of clinical information and should be stored for a period of 11 years to meet medico-legal requirements and function as aids for clinical research and teaching [1]. The British Orthodontic Societyrecommends that dental plaster casts should be retained for 11 years or until young patients reach 26 years of age[1].This will result in an accumulation of plaster casts that need more space for storage and more time consuming in cast retrieval. Three-dimensional virtual dental models represent an alternate, convenient way of recording and maintaining occlusal information on hard drives [2,3].

To solve this problem, many new technologies have been developed, such as reconstructing virtual models of dental casts using mechanical probes[4,5]and optical lasers[6,7,8,9]instead of taking manual measurements. For example, 3D digital models of scanned dental plaster casts were reconstructed and measured using a line laser scanner. When compared with measurements of the actual models, the difference was within 0.3 mm[10].

There are several advantages to using these technologies, including measurement accuracy, saved time and reduced requirements for physical storage space [11]. Currently, a few clinically operational 3D technologies can provide accurate recording and measurements from dental plaster casts as a replacement for manual measurements [6,12,13].However, these technologies require expensive hardware devices and system-specific software that are not suitable for primary hospitals and individual clinics.

This study aims to design a feasible, efficient, low-cost method for reconstructing the 3D models of orthodontic plaster casts. Multi-baseline digital close-range photogrammetry (MBDCRP) is a mature technology to acquire 3D geometric information for real-world objects from stereoscopic image and has potential application in orthodontics to record plaster casts three-dimensionally. With a holographic sensor SLR digital camera, photos of dental casts were taken using a multi-short baseline method. The images were then imported into a programme designed for computerized space analysis. The 3D morphology of the dentition was then reconstructed. Numerical analyses of the morphology, such as of the mesio-distal crown diameter, arch width, and arch perimeter, supplied objective information about each dental cast.

To evaluate the reliability of the MBDCRP, photogrammetric measurements were compared with calliper measurements of the same study casts, which served as a gold standard [14].

## Materials and methods

### Individual dental cast reconstruction

**Fig 1:**
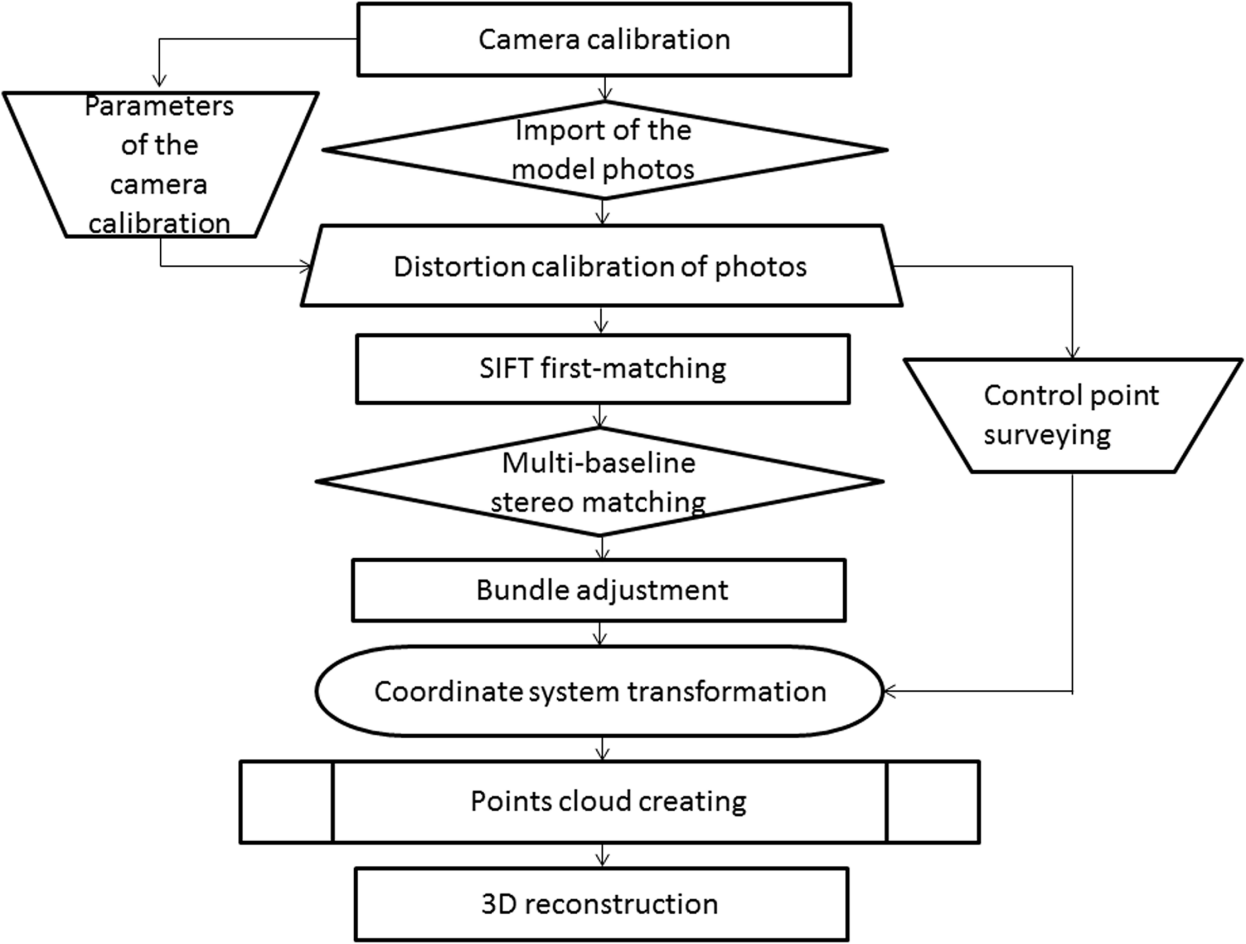
Schematic illustration of the 3D model reconstruction procedures.

A flowchart for reconstructing a 3D dental model based on MBDCRP was shown in Fig 1. This system consisted of 5 stages: (1) image acquisition, (2) camera calibration, (3) multi-baseline stereo matching, (4) calculation of photo parameters, and (5) 3D reconstruction.

### Sample description

Thirty sets of dental plaster casts were randomly selected from the Department of Orthodontics, Affiliated Hospital of Stomatology, Chongqing Medical University. Each study model was cast into a standard form without voids and fractures and uniformly polished with its base parallel to the occlusal surface. All models clearly recorded the complete dentition, basal bone, mucosal turning, frenum, palatal rugae, and other anatomical structures. All teeth had normal morphology without heavy abrasions, defects. Besides that horizontal overlaps between any two teeth were not more than 2 mm. All measurements were carried out both manually and digitally on these models.

### Image acquisition

**Fig 2:**
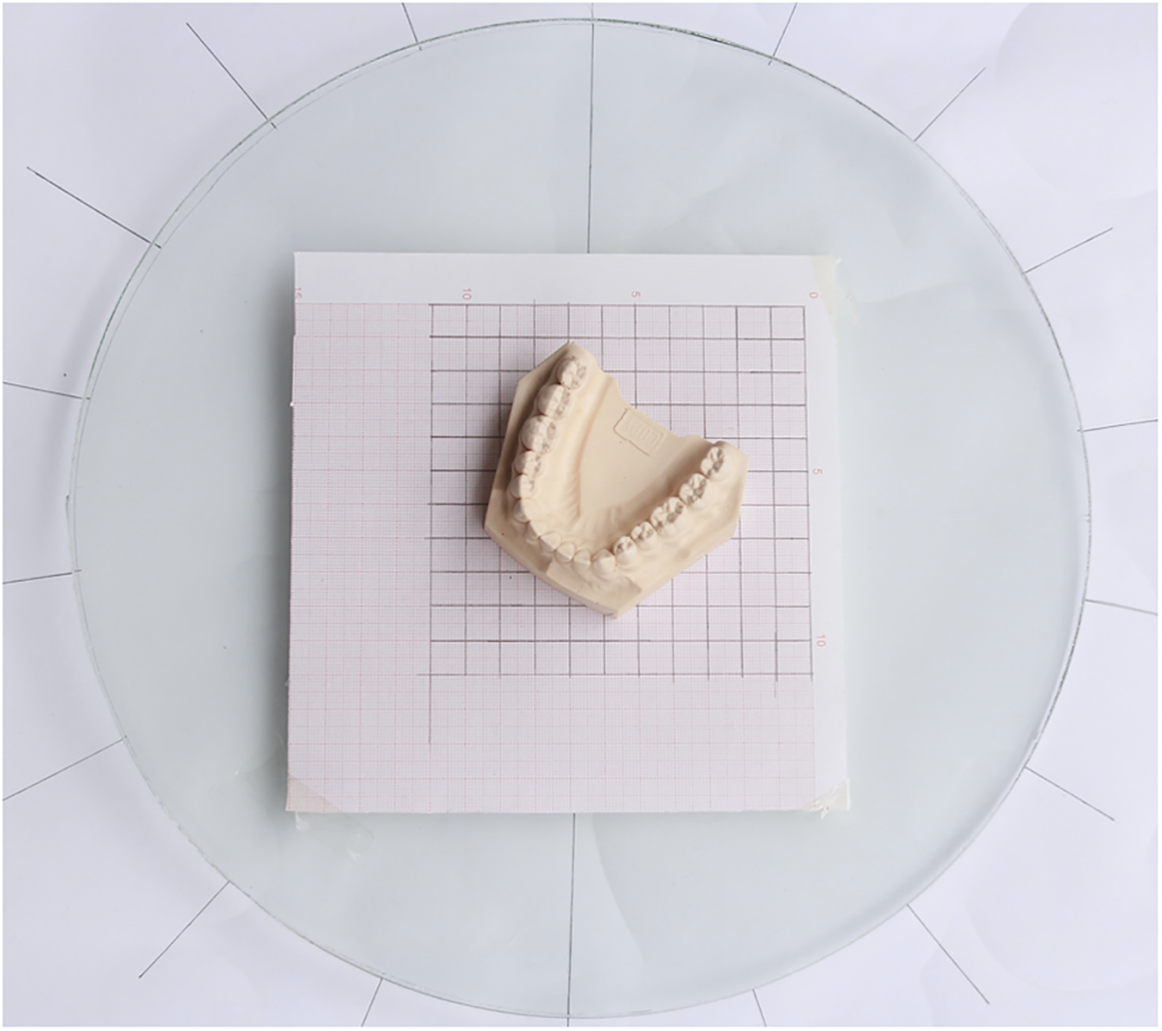
Homemade studio for dental casts photography.

An SLR digital camera (Canon EOS 600D) with a 90 mm prime lens (Teng long Company) was used in this research. A 10*10 mm grid coordinate was used as the control points and control field for the analytical calculations and was pasted on a glass surface that served as a platform, which was made in house (Fig 2). The dental plaster casts were placed on the platform. The camera was fixed on a tripod with a wide aperture (f = 22) and specific object distance (45 cm). Camera resolution was 3456×2304 square pixels. The camera was used to take photos around the platform with each rotation of 20°, from 0° and 40° angles overhead. In total, 72 photos were taken of each dental cast.

### Camera calibration

A calibration of the intrinsic parameters (radial and tangential distortions, coordinates of the principal points, and focal length) of the camera was needed [15]. A method based on a dot grid of an LCD screen was used by taking pictures of the upper left, upper right, lower right, and lower left corners of the LCD screen horizontally and vertically, totalling eight photos [16]. The camera’s intrinsic parameters were computed by correlating the coordinates of the known dot grid with the corresponding coordinates on the photos using the Camera Calibration Toolbox for MATLAB [17].

### Multi-baseline stereo matching

A matching strategy that took advantage of the short baseline and the multiple photos of the dental casts was used to avoid large image distortions. This approach had proven very useful in solving the image matching problem [18,19,20]. Because neighbouring photos were taken at a short baseline, there were almost no gross distortions between these photos and a great many overlaps between the images. There were many corresponding points for each photo, which helped in making automatic matches according to the corresponding points in neighbouring photos.

### 3D reconstruction

**Fig 3:**
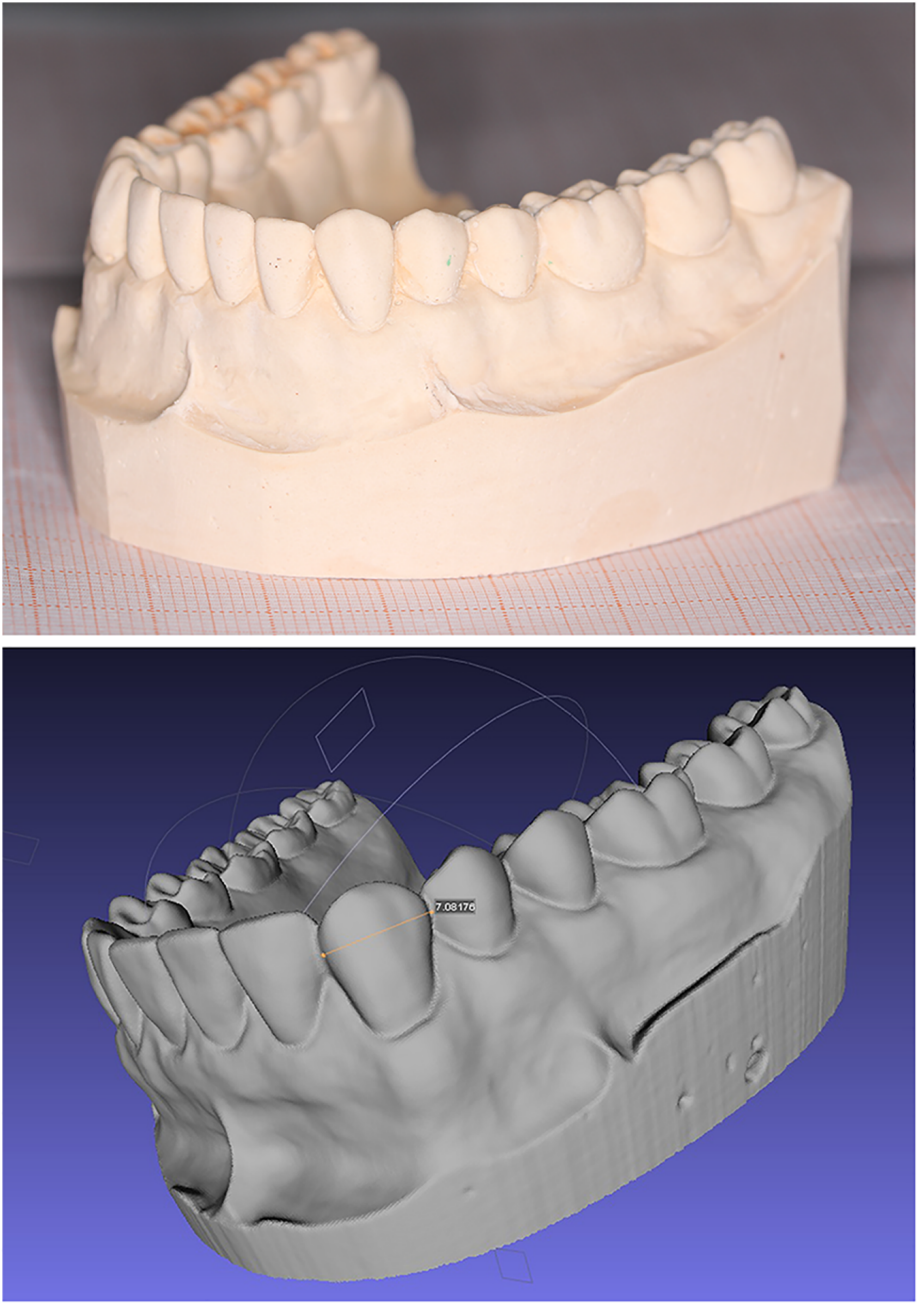
A dental cast and the 3D digital model of reconstruction.

After the image matching process, 3D points were calculated using the image matching results; these were known as point clouds. The 3D points contained the three-dimensional coordinate information of the corresponding points [21]. Using the open source software MeshLab, these point clouds were used to build triangular networks, three-dimensional textures and veneers. Finally, the 3D models of the dental casts were visualized and reconstructed (Fig 3).

### Measurements of the models

Two examiners with prior training carried out all the measurements of the 3D virtual models and the dental casts independently. All the measurements were repeated six times, with a one-week interval between measurements.

**Fig 4:**
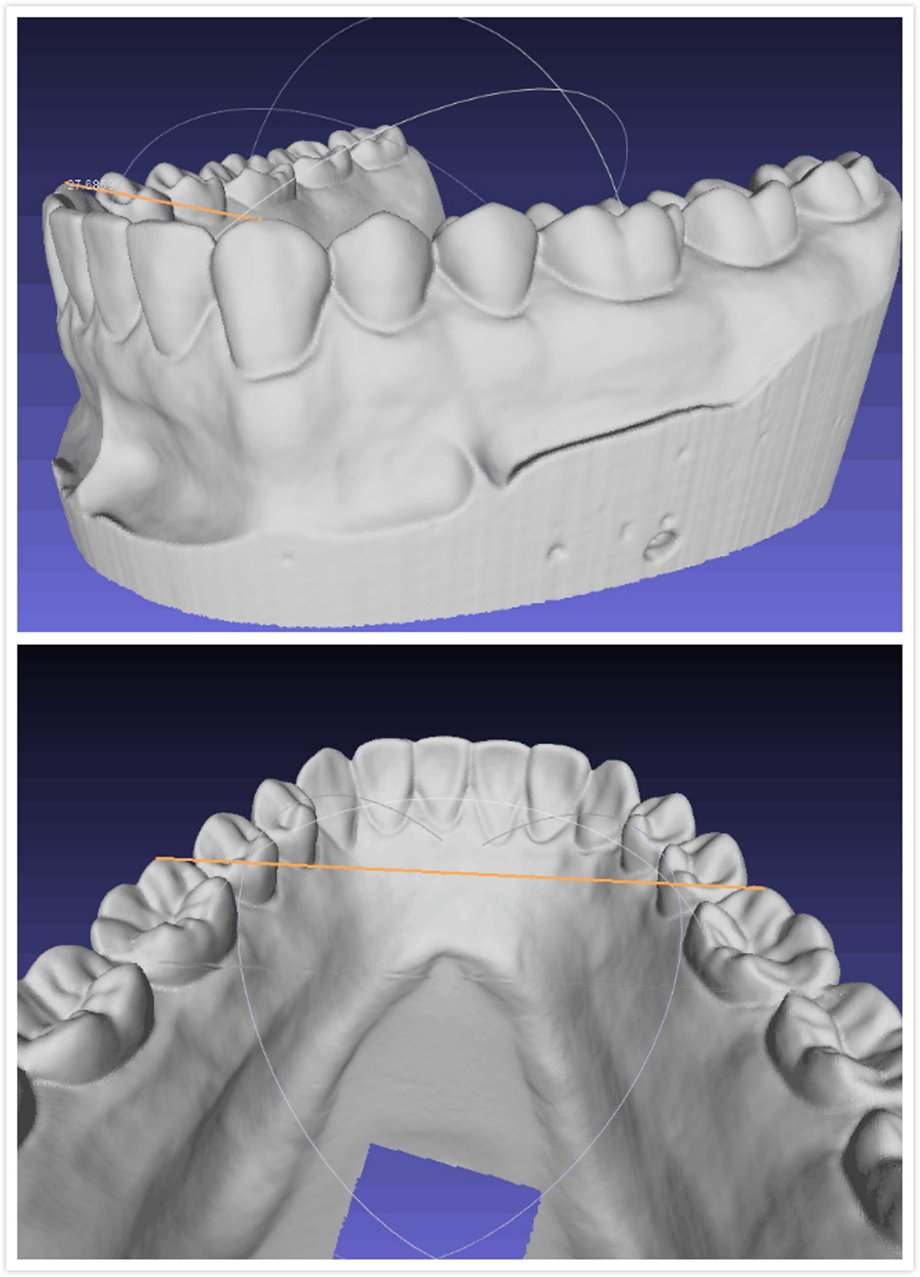
Measurements of intercanine and intermolar width using MeshLab.

The digital models could be rotated and magnified for point identification. Measurements were taken from point to point with an accuracy of 0.001 mm. However, all values were rounded to the nearest 0.01 mm. The mesio-distal widths of the upper right central incisors (UR1), upper left canines (UL3), and lower left first molars (LL6) were measured by dragging a line between the reference points using a computer mouse from the occlusal view. The upper and lower intercanine widths (UICW, LICW) and interfirst molar widths (UIM1W, LIM1W) were measured respectively from the cusp tips of the canines and the mesial buccal tips of the first molars (Fig 4). The upper and lower arch perimeters (UAP, LAP) were measured from the distal of the second molar to its counterpart around the arch.

In the same way, manual measurements were performed directly on the plaster casts using a digital calliper equipped with a resolution of 0.01 mm (Mitutoyo, type DP-15DC, UK).

### Statistical analysis

All measurements were recorded and saved in a Microsoft Excel 2003 spreadsheet (Microsoft, Redmond, Wash). The intraclass correlation coefficient (ICC) statistic was used to assess the intra- and interexaminer reliability of the digital and manual measurements. After determining the interexaminer reliability, all the parameters measured by the 2 examiners were pooled. The means for each parameter and for each method were calculated and compared between the digital and plaster models. To analyse the degree of agreement between the digital and manual measurements, the Bland–Altman-method was used [22].

## Results

The 3D dental models were reconstructed using MBDCRP. Each digital model clearly showed the morphologies of the tooth, lip, cheek, frenulum linguae, mucosal folds and other anatomical structures. Compared with the plaster casts, the cusps, nests, trenches, and ridges could be distinctly recognized. The dentition characteristics of the digital models were consistent with those of the plaster casts (Fig 3).

For all evaluated parameters, the differences between the measurements of examiners 1 and 2 are shown in Table 1. The differences ranged from –0.199 to +0.273 mm for the calliper measurements of the dental casts and from −0.669 to +0.026 mm for the digital models. The coefficient of variance (CV) ranged between 0.235–6.731% and 0.219%–7.121% for the repeated direct measurements and between 0.165%–5.898% and 0.242%–6.731% for the repeated digital measurements. The interexaminer correlation coefficients (ICCs) ranged from 0.908 to 0.999 for the calliper measurements and from 0.879 to 0.998 for the digital measurements, indicating good intrarater reliability. The analytical results showed that each method had high reproducibility for all variables and low variation between the two examiners.

**Table 1:** Interexaminer agreement showing the difference of the means made by the two examiners on casts and digital models expressed as coefficient of variation (CV(%)) and interclass correlation coefficient (ICC) (n = 30).

Parameter	Examiner1 cast	Examiner 2 cast	difference	ICC(95%CI)	Examiner 1 digital	Examiner 2 digital	difference	ICC (95%CI)
	CV(%)	CV(%)			CV(%)	CV(%)		
UR1	3.776	3.876	-0.029	0.990 (0.969 to 0.996)	3.778	3.704	0.002	0.996(0.989 to 0.998)
UL3	0.235	0.219	0.003	0.908 (0.748 to 0.968)	0.165	0.242	0.007	0.879(0.640 to 0.959)
LL6	4.680	4.653	0.007	0.999(0.997 to 0.999)	4.602	4.621	-0.004	0.998(0.996 to 0.9999)
UICW	5.905	5.470	-0.189	0.965 (0.898 to 0.988)	5.898	5.926	0.008	0.994(0.990to 0.9995)
UIM1W	2.053	1.967	0.035	0.931 (0.807 to 0.976)	2.336	2.150	-0.330	0.921 (0.765 to 0.974)
UAP	4.857	4.678	0.273	0.964 (0.895 to 0.988)	4.477	4.473	-0.669	0.908 (0.890 to 0.939)
LICW	3.721	3.332	-0.199	0.923 (0.786 to 0.973)	3.730	3.735	0.019	0.938(0.912 to 0.953)
LIM1W	6.731	7.121	-0.101	0.994 (0.983 to 0.998)	5.839	6.731	-0.107	0.926(0.901 to 0.946)
LAP	5.497	5.249	-0.158	0.996 (0.988 to 0.998)	5.401	5.435	0.026	0.928 (0.905 to 0.955)

ICC Values closest to 1.00 are most reproducible. Coefficient of variation, CV (%) = (SD/mean)×100. UR1,upper right central incisor;UL3,upper left canine;LL6,lower left first molar; UICW, upper intercanine width; UIM1W, upper interfirst molar width; UAP, upper arch perimeter; LICW, lower intercanine width; LIM1W, lower interfirst molar width; LAP, lower arch perimeter.

A comparison of the measurements made on the plaster casts and the 3D digital models by examiner 1 is presented in Table 2. The mean difference between measurements ranged between 0.011 and 0.402 mm, which indicated that the values obtained from digital models were slightly smaller than those from plaster casts. These differences were not statistically significant except for LAP (*P<*0.05), and all the differences were regarded as clinically acceptable (<0.5 mm).

**Table 2:** Intermethod agreement showing the standard deviations (SDs) of the means (d) and *P* value of measurements made by the two examiners on casts and digital models (n = 30).

Parameter	Measurement(mm)		Measurement(mm)		Mean(d)	SD	*P* value	d+1.96*SD	d-1.96*SD	%ofvalues±2SD*
	Cast		Digital							
Mean	SD	Mean	SD						
UR1	8.451	0.315	8.440	0.315	0.012	0.009	0.082	0.029	-0.005	100
URL3	8.172	0.022	8.162	0.018	0.011	0.007	0.062	0.023	-0.002	100
LL6 UICW	10.958	0.498	10.943	0.494	0.016	0.011	0.158	0.037	-0.006	100
	36.751	1.722	36.643	1.722	0.109	0.080	0.071	0.270	-0.052	100
UIM1W	50.253	1.155	50.099	1.182	0.154	0.209	0.069	0.561	-0.261	96.67
UAP	103.041	4.842	102.721	4.517	0.316	0.995	0.236	2.273	-1.643	96.67
LICW	29.166	1.627	29.044	1.653	0.122	0.239	0.093	0.592	-0.350	96.67
LIM1W	38.880	2.631	38.776	2.402	0.108	0.169	0.169	0.931	-0.722	93.33
LAP	97.566	4.966	97.165	5.173	0.402	0.632	0.002	1.642	-0.841	93.33

Mean (d) represents mean difference between methods, Mean(d) = Cast(mean)-Digital(mean) *P* value represents the significance of the paired t-test

*The value refers to the percentage of measurements lying in 2 SD of the mean difference. UR1, upper right central incisor; UL3, upper left canine; LL6, lower left first molar; UICW, upper intercanine width; UIM1W, upper interfirst molar width; UAP, upper arch perimeter; LICW, lower intercanine width; L IM1W, lower interfirst molar width; LAP, lower arch perimeter.

**Fig 5:**
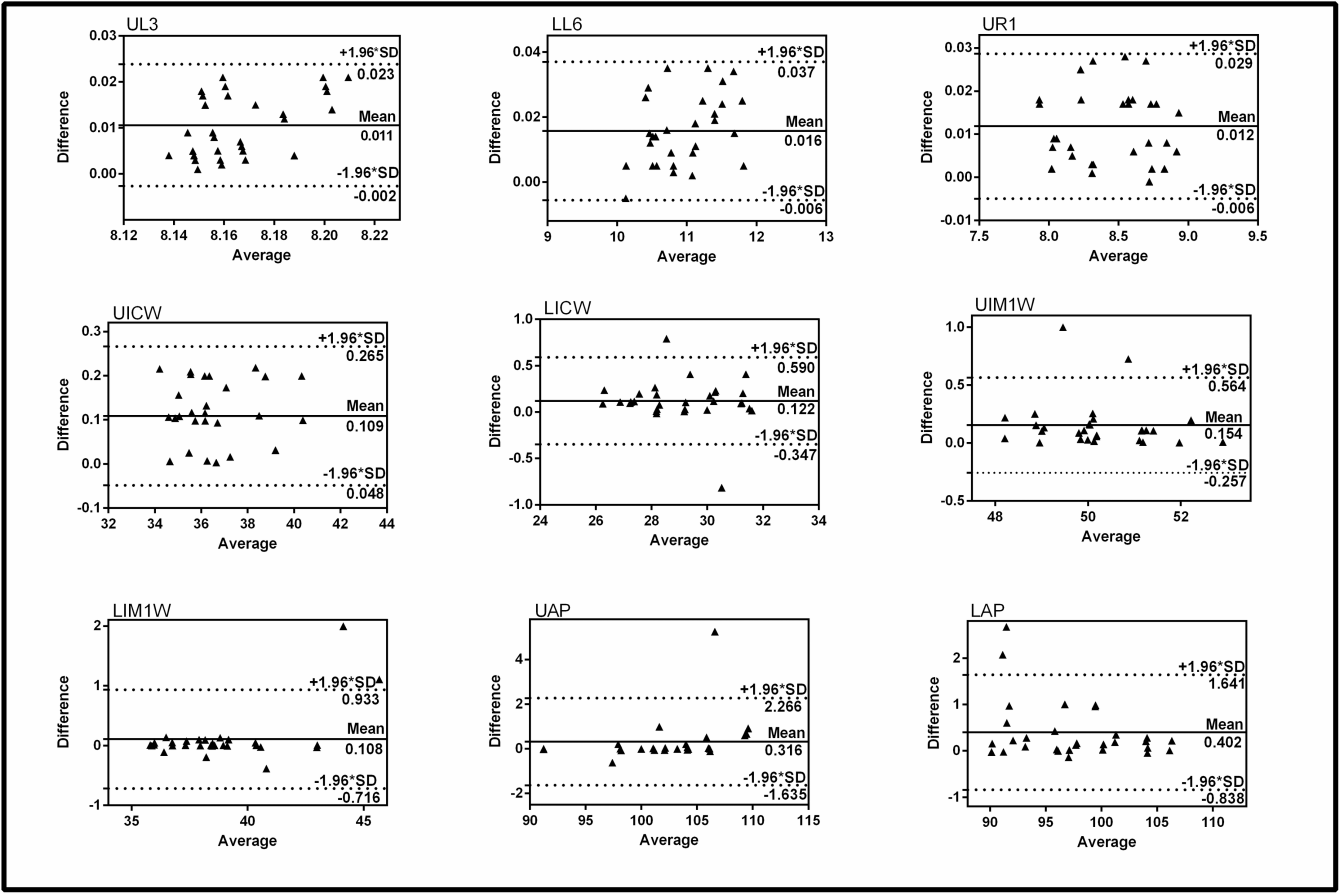
Bland-Altman plots comparing the measurements obtained by caliper-based and photogrammetric 3D digital measurement.

The Bland–Altman analysis revealed a range that accommodated 95% of the differences between any of the values yielded by the different methods. The mean differences and limits of agreement resulting from our comparison of the two measuring techniques (calliper-based and MBDCRP-based) are summarized in Fig 5. All the differences were clustered within the mean difference ±2SD for UL3, UR1, LL6and UICW, indicating good coincidence between these two methods. However,96.67 per cent of the values were within the mean difference ±2SD for UAP,UIM1W and LICW, falling within−1.643to +2.273mm, -0.261 to +0.561mm, -0.350 to +0.592mm, respectively. For LAP and LIM1W, 93.33 per cent of the values were within the mean difference ±2 SD, rangefrom−0.841to+1.642mm,and -0.722 to + 0.931mm.Thus, at least 93 per cent of these differences were within the mean difference ±2SD for all measured parameters.

## Discussion

In the present study, we analyzed 3-D models of dental plaster casts, using MBDCRP, which was a well-established technique for 3D reconstruction of real objects from two-dimensional image overlap and making measurements from photographs [23]. Sometimes, this type of photogrammetry was also called Image-Based Modeling. Along with the advance of some open-source software, off-the-shelf digital cameras could be used to extract highly accurate 3D models of real- objects. It had been widely applied to model and measure buildings, engineering structures, mines, earth-works, stock-piles, etc. In this study, the outputs of photogrammetry were 3D model of orthodontic casts. They were reconstructed clearly. All the anatomical structures like cusps, nests, trenches, and ridges were consistent well with those of the plaster casts (Fig 3). From this, we could deduce that the resolution of photos (3456×2304 pixels) were suitable for 3D reconstruction of dental casts. Furthermore, all the study models were selected from clinical cases that exhibited Class I, II, and III malocclusions without severe horizontal overlap. Therefore, this research was applicable to all cases encountered in clinical practice except for severe horizontal overlap(>2mm) between any two teeth.

Traditionally, the measurements made directly on the casts using electronic callipers were considered to be the gold standard. Until now, it is still widely used to measure and analyse dental casts clinically. However, this technique depended on locating the calliper on a given mark and reading the distance from the ruler on the calliper manually [24]. Consequently, the technique was subject to intra- and inter-operator variation, which was time consuming and inefficient. When examiner 1 measured the casts manually six times, the CVs of the measurements ranged from 0.235% -6.731%. The CVs of the measurements of examiner 2 were within a range of 0.219% to 7.121%. The intraclass correlation coefficients [25] for interexaminer reliability were 0.908–0.999. These results demonstrate that all intra- and interexaminer measurements had excellent reproducibility.

Nevertheless, digital models, such as the 3D reconstructions of manually created models, have been reconstructed using many methods, such as laser scanning. Correia GD et al.[26] reported that digital models obtained using a 3Shape R700T scanner were as accurate and reproducible as plaster casts; the differences between the two methods were within clinically acceptable limits. Using a similar method, 3D digital models were formed by converting manual plaster casts into digital models by means of multi-baseline digital close-range photogrammetry technology. When the measurements were made on the 3D digital models, the CVs of the measurements of examiners 1 and 2 ranged from 0.165%–5.898% and 0.242%–6.731%, respectively, which were smaller than the manual measurement variations.

The need for an operator to manually click on points with a mouse for measurement was the main source of operator-associated variation. Because the light from shooting the photos travelled in straight lines and the back surfaces were obscured, the marked points on the 3D models were not as clear and sharp as those on the plaster casts, especially on the proximal surfaces of the teeth. To reduce this problem, the camera was moved around each dental cast in different angulations during the process of image acquisition. The data from each photo were registered and merged together. In addition, because a region of interest could be chosen in each digital model and enlarged and because the digital measurements were directly readable, the use of the photogrammetry technology contributed to the accuracy of the results. Random errors were also reduced by replicating the measurements six times and averaging the results. The 3D model measurements that were obtained using the photogrammetry technology had higher repeatability than the traditional manual calliper method. This result is consistent with a study by Bell A. et al.[24], which reported that the overall mean difference in digital measurements was slightly smaller than that obtained from manual measurements.

The standard deviations associated with the differences between repeated measurements were used to assess reproducibility. When comparing the measurements obtained through the two methods, the reproducibility for each measured parameter was different, which may be due to the span between the marked points and the complex topography. In this study, the greatest difference for the lower arch perimeter was 0.402 mm (SD = 0.632 mm) and was obtained from digital versus calliper-based measurements. This difference was statistically significant. A previous study by Leifertet al. [27]reported that although a statistically significant difference in maxillary arch length of less than 0.50 mm could be measured, the difference was not clinically significant. Okunami et al. [28]also found that a mean difference of 0.5 mm between digital and plaster dental casts seemed to have no practical consequences; such a difference is too small to be noticed clinically and thus may not be clinically significant. Thus, differences of less than 0.5 mm for single tooth measurements and less than 5 per cent of the distance of measurements of arch width, arch length, and arch perimeter were postulated as the limits of clinical acceptability[29]. The difference of the lower arch perimeter was also regarded as having no clinical implications in this study. The systematic difference in lower arch length was most likely due to crowded dentition, accumulated distortions within pictures and the large-scale reconstruction employed. The differences between the measurements of mesio-distal tooth width and arch width for select teeth were within a smaller range of 0.011 to 0.016 mm and 0.108 to 0.154 mm. The measurements of tooth width and arch width (intercanine width and intermolar width) were the most reproducible and accurate, whereas the lower arch perimeter was found to have large variation and poor reproducibility. Furthermore, the digital measurements were found to be smaller than the calliper measurements, which is similar to results obtained by Johanna Radeke et al. [30] using OnyxCeph3^TM^ analysis software.

In addition, the Bland-Altman limits of agreement technique is an effective approach for assessing consistency between different methods and showed that the differences in the measurements of the plaster and 3D models were all clustered around the mean difference. In particular, they were within two standard deviations (2SD) of the mean difference for UL3, UR1, LL6, UICW, which indicates good intra-rater reliability. However, even for the maximum deviation of LAP, which fell between −0.841 and +1.642mm, 93.33 per cent of the values were within the mean difference ±2 SD, and the precision ranges for the upper and lower limits were narrow, indicating that the actual limits of agreement were precise and that the values obtained from the two methods were in agreement. With this method, 3D models seem to be a viable alternative for analysing study models, as well as for orthodontic diagnosis and treatment planning.

This method, which has the advantages of reduced cost, savings in storage space, and the ability to share digital data online, may be beneficial for orthodontists from both the management and marketing perspectives. However, measurements taken using photogrammetry technology are not suitable for dentition with severe horizontal overlap between any two teeth. The next step, the development of new techniques like time-of-flight 3D cameras, mirror systems, and allreflective optical systems, from manual image measurement to fully automatic 3D model reconstruction and measurement will be applied in MBDCRP process. It will be a more effective way in the clinical use of storage, measurement and analysis of dental casts, including severe horizontal overlap ones. Furthermore, reconstruction the occlusal relationship by unifying the world coordinate system of maxilla and mandible, MBDCRP will be practically used in clinical diagnosis of malocclusion and treatment planning.

## Conclusions

This study showed that 3D models of dental casts without severe horizontal overlap were reconstructed by a novel method of MBDCRP successfully.

- The measurements made on 3D digital models were reproducible.
- The differences between measurements made on 3D digital models and plaster casts were not statistically significant except for LAP. The mean difference of ALP was much less than 5 per cent of the distance of arch length, also regarded as having no clinical significance.
- Without expensive hardware and software, MBDCRP is an alternate way to store and measure dental models without requiring a special room to keep plaster casts, especially suitable for private dental clinics and community clinics.

## Supporting information

S1 Table All the parameter values for digital versus cast-based measurements. (XLSX)
